# Satisfaction with Fertility Preservation Decisions among Adolescent Males with Cancer: A Mixed Methods Study

**DOI:** 10.3390/cancers13143559

**Published:** 2021-07-16

**Authors:** Charleen I. Theroux, Kylie N. Hill, Anna L. Olsavsky, James L. Klosky, Nicholas D. Yeager, Anthony Audino, Sarah H. O’Brien, Gwendolyn P. Quinn, Cynthia A. Gerhardt, Leena Nahata

**Affiliations:** 1 Center for Biobehavioral Health, The Abigail Wexner Research Institute at Nationwide Children’s Hospital, Columbus, OH 43205, USA; Charleen.Theroux@nationwidechildrens.org (C.I.T.); Kylie.Hill@nationwidechildrens.org (K.N.H.); Anna.Olsavsky@nationwidechildrens.org (A.L.O.); Sarah.Obrien@nationwidechildrens.org (S.H.O.); Cynthia.Gerhardt@nationwidechildrens.org (C.A.G.); 2 Department of Pediatrics, Emory University School of Medicine and Aflac Cancer and Blood Disorders Center, Children’s Healthcare of Atlanta, Atlanta, GA 30329, USA; james.klosky@emory.edu; 3 Division of Hematology/Oncology, Nationwide Children’s Hospital, Columbus, OH 43205, USA; nicholas.yeager@nationwidechildrens.org (N.D.Y.); anthony.audino@nationwidechildrens.org (A.A.); 4 Department of Pediatrics, The Ohio State University College of Medicine, Columbus, OH 43210, USA; 5 Department of Obstetrics and Gynecology, New York University Grossman School of Medicine, New York, NY 10016, USA; Gwendolyn.Quinn@nyulangone.org; 6 Division of Endocrinology, Nationwide Children’s Hospital, Columbus, OH 43205, USA

**Keywords:** fertility preservation, sperm banking, adolescent and young adult oncology, decisional satisfaction

## Abstract

**Simple Summary:**

Fertility impairment is common among male childhood cancer survivors and negatively impacts quality of life. Sperm banking, before starting cancer treatment, is an established fertility preservation option, yet it remains underutilized at many pediatric centers. Although survivors often report regret about missed banking opportunities, little is known about short-term decisional satisfaction. The aim of this mixed methods study was to examine decisional satisfaction one month after diagnosis by comparing satisfaction among families of adolescents who did or did not attempt to bank. Quantitatively, families reported satisfaction regardless of the banking decision, while qualitatively, families of adolescents who did not attempt to bank reported potential for future regret. Thus, decisional dissatisfaction may not present after one month but could emerge in the future. The findings underscore the importance of longitudinal research to examine satisfaction over time, and why quantitative and qualitative discrepancies exist, as well as psychosocial support across the care continuum as survivors approach their reproductive years.

**Abstract:**

Half of male childhood cancer survivors experience treatment-related fertility impairment, which can lead to distress. Survivors often regret forgoing fertility preservation (FP), and decisional dissatisfaction is associated with a lower quality of life. This mixed methods study examined short-term FP decisional satisfaction among families of male adolescents newly diagnosed with cancer who received an initial fertility consult and completed an FP values clarification tool. One-two months after the FP decision, thirty-nine families completed the Brief Subjective Decision Quality measure. Decisional satisfaction was compared for participants (mothers, fathers, adolescents) who did and did not attempt to bank. Semi-structured interviews included the following question: How do you/your family feel about the banking decision now/in the future? Decisional quality scores were moderate-high (*M* = 5.74–6.33 out of 7), with no significant differences between non-attempter (*n* = 15) and attempter (*n* = 24) families (adolescents: *p* = 0.83, *d* = 0.08; mothers: *p* = 0.18, *d* = 0.45; fathers: *p* = 0.32, *d* = 0.44). Three qualitative themes emerged among non-attempter families: (1) satisfaction with decision (50% of participants), (2) acceptance of decision (60%), and (3) potential for future regret (40%). Satisfaction with decision was the only theme identified in attempter families (93%). Quantitively, short-term decisional satisfaction was high regardless of the banking attempt. However, the qualitative findings suggest that the experiences of families who did not bank may be more nuanced, as several participants discussed a potential for future regret, highlighting the importance of ongoing support.

## 1. Introduction

With improvements in cancer care and treatment methods, more children and adolescents are surviving cancer and entering adulthood [1,2]. Unfortunately, many of these survivors will experience late effects from cancer and its treatment. For example, approximately half of male survivors experience fertility impairment secondary to gonadotoxic therapy [3]. Infertility can lead to psychosocial distress, as many adolescents with cancer desire biological parenthood, and the inability to achieve these goals is associated with a lower quality of life [4,5,6,7]. In a recent study of adolescent males newly diagnosed with cancer, parenthood was viewed as a “top 3” life goal [8]. In an additional study, 80% of cancer survivors desired biological children, and two thirds noted that they would feel distress if this was unattainable [5].

The ability to achieve biological parenthood can be safeguarded through the use of fertility preservation (FP). Sperm banking prior to treatment initiation is a safe and effective way for pubertal males to protect their ability to have biological children [9]. Fertility consultations by trained medical professionals have been increasingly incorporated into standard care to ensure that families are provided with adequate education surrounding adolescents’ level of infertility risk and appropriate FP options [10,11]. Despite the expansion of these services, sperm banking rates remain around 50% or less at many centers [12].

The family plays a key role in adolescent FP decision making. According to family systems theory, individuals are interconnected to their family context, with family members interacting to influence each other’s behavior [13,14]. Thus, in the context of a new cancer diagnosis, members of the family are affected and involved in making healthcare-related decisions. Along these lines, recent studies among male adolescents with cancer have identified parents, and particularly fathers, as key influencers in sperm banking decision making [15,16,17]. Due to their young age, adolescents’ future-oriented thinking may be developmentally limited, further highlighting the importance of family-centered research on the decision making experiences of this population [18].

Studies of cancer survivors have shown that survivors’ parenthood goals and preferences can shift as one moves from adolescence to young adulthood, which can contribute to decisional regret not only in patients/survivors who decided to forgo FP but also among their parents [4,6,19]. Therefore, research focused on both parent and adolescent perspectives is warranted, especially among younger adolescent populations where a parent may play a more active role in decision making [20]. Overall, high decisional regret is associated with a reduced quality of life [21,22]. Additionally, high decisional regret in the long term, as well as high decisional conflict in the short term [19,23,24,25], can have a negative impact on physical and mental health [26,27,28]. However, timely and comprehensive FP counseling has been associated with lower decisional conflict [23,24], lower decisional regret [29], higher banking rates [25], and higher quality of life [30]. Furthermore, the use of decision tools/aids has also been linked to lower regret and higher banking rates [31,32]. Taken together, the use of quality fertility counseling and decision tools may mitigate short-term decisional conflict and long-term decisional regret, thus improving quality of life for patients and their families.

While most research has focused on the experiences of adults [4,6,19] and/or females [23,24,25] with cancer, less is known about how families of adolescent males with cancer feel about these decisions in the short term. Exploring short-term decisional satisfaction is especially important because identifying when regret may begin to occur can inform clinical interventions to prevent distress in the long-term and enhance quality of life throughout the treatment continuum. The aim of this study was to use a mixed methods approach to examine decisional satisfaction about FP among adolescent males newly diagnosed with cancer and their parents.

## 2. Methods

### 2.1. Procedure

Both quantitative and qualitative data were gathered as part of a larger institutional review board-approved study examining the impact of a novel values clarification tool on FP decision making in male adolescents newly diagnosed with cancer. Adolescents and their parents (up to two caregivers) were enrolled following a fertility consult performed by a member of the clinical team. This study took place at a site with an established pediatric FP program with an opt-out model [12], meaning that a consult on fertility and reproductive health is automatically placed for all patients at the time of a new cancer diagnosis. During the consult, infertility risk was reviewed, and an overview of appropriate FP options was provided. Our study team collaborated with members of the clinical team (patient’s oncologist and fertility navigator) to determine eligibility for the study. Inclusion criteria were: (a) male; (b) 12–25 years of age at the time of diagnosis; (c) scheduled to receive chemotherapy and/or radiation as a treatment regimen; (d) pubertal/eligible for sperm banking (i.e., at least Tanner stage 2–3 as determined during fertility consult); (e) developmentally/cognitively able to complete the study; and (f) fluent in English.

**Figure 1:**
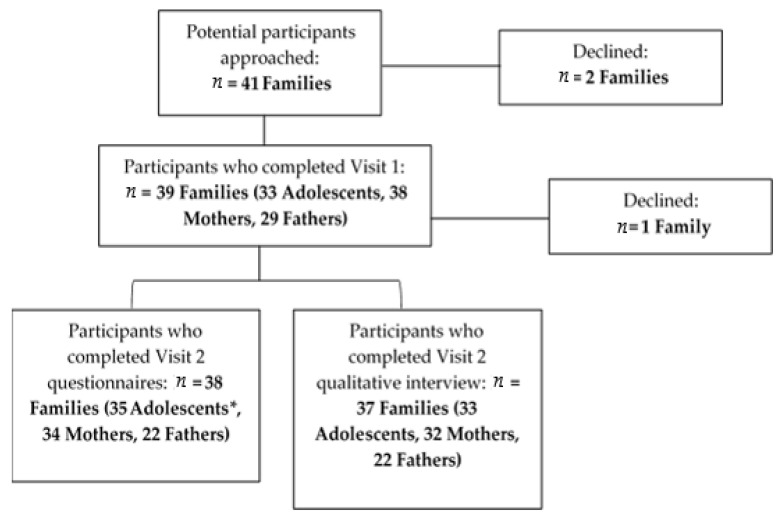
Recruitment flowchart. Participation across study time points. * Three adolescents completed Visit 2 but did not complete Visit 1.

Visit 1. Families were approached in the clinic or the hospital after diagnosis and following the fertility consult, but before cancer treatment began (see Figure 1). Study staff recruited families and obtained consent and/or assent (if applicable). Families completed a demographic questionnaire followed by an FP values clarification tool (Family-centered Adolescent Sperm banking values clarification Tool, or FAST) [31]. Study staff also gathered information on adolescents’ diagnosis, treatment plan, and fertility risk from medical records utilizing a standard medical abstraction form. Following Visit 1, families were provided with a USD 5 meal card.

Visit 2. One to two months following the completion of Visit 1, families were approached in person or over the phone to examine their FP decision-making process. This time point was chosen to ensure recovery from the first cycle of chemotherapy while maximizing families’ recall of their FP conversations and decision making [33,34]. During this visit, families were asked to complete the Brief Subjective Decision Quality (BSDQ) measure [35]. This questionnaire required participants to self-report ratings (ranging from 1–7, with the added option of “Not Offered”) on six items related to decisional quality: regret, satisfaction, and fit, as well as perceived adequacy of information, time, and involvement. Scores on these individual items were combined to create a composite decision quality score. Additionally, trained research assistants conducted one-on-one semi-structured interviews, with parents and adolescents separately, to examine the FP decision-making process. This study focused on data derived from the following questions: How do you feel about the decision you made about whether or not to bank sperm? How do you think you (your family) will feel about this decision when you are older? Caregivers were asked an additional question to capture their perspectives in relation to their sons: How do you think you will feel about this decision when your son is older? How do you think your son will feel about this decision when he is older? How do you think your family will feel about this when your son is older?

### 2.2. Quantitative Analysis

Quantitative data were analyzed using SPSS Statistics version 25.0 (IBM SPSS Statistics for Windows, Version 25.0. Armonk, NY, USA: IBM Corp) [36]. Descriptive statistics (e.g., *M*, *SD*, range, frequencies) summarized sample characteristics, and the independent samples *t*-test (α = 0.05; two-tailed) compared BSDQ scores by banking attempt group (attempters vs. non-attempters) for each participant type (adolescent, mother, father). The paired samples *t*-test compared BSDQ score by participant type (adolescent, mother, father).

### 2.3. Qualitative Analysis

Interviews were audio recorded and transcribed verbatim by members of the study staff. Transcripts were separated into two categories based on whether the adolescent had attempted FP. Adolescents, mothers, and fathers were included in both groups. Transcripts were reviewed by three members of the study team (C.I.T., K.N.H., L.N.), with input from C.A.G. and A.L.O. Thematic content analysis was conducted using the constant comparative method and inductive identification of themes [37]. In this process, coders reviewed transcripts using NVivo (QSR International, Burlington, MA, USA) in groups of 5 and identified any emergent themes and codes. The non-attempter group was coded first followed by the attempter group. Saturation was reached with 7 participants for the non-attempter group and 5 participants for the attempter group. All transcripts were reviewed, and frequency counts were then identified for each theme.

### 2.4. Mixed Methods Approach

A mixed methods approach was chosen because it allowed for the integration of self-reported satisfaction while also allowing families to discuss their experiences in their own words. Both quantitative and qualitative data were analyzed separately, consistent with a convergent parallel mixed methods design [38]. Results were then compared and integrated.

## 3. Results

### 3.1. Participants

Of the 41 families approached, 39 families had at least one member (i.e., mother, father, and/or adolescent) enroll in this study. Thirty-five adolescents (age range: 12.33 to 20.75 years, *M* = 16.29, *SD* = 2.25 years, see Table 1), 34 mothers, and 22 fathers completed the Visit 2 questionnaires, and 33 adolescents, 32 mothers, and 22 fathers completed the qualitative interviews. Of the 39 families enrolled in this study, 62% (*n* = 24) of adolescents made a banking attempt. Most participants who banked provided a sample through masturbation, while one participant underwent testicular sperm extraction, and an additional participant made a banking attempt but was unsuccessful in producing a sample.

### 3.2. Quantitative Results

Decisional quality scores were moderate-high (*M* = 5.74–6.33 out of 7; see Table 2). Paired samples *t*-tests revealed that the father composite BSDQ score (*M* = 6.37) was significantly higher than the patient BSDQ score (*M* = 5.93; *t*(19) = −2.260; *p* = 0.036; see Table 3). In quantitative analyses, there were 23 adolescents, 21 mothers, and 12 fathers in the attempter group, and 12 adolescents, 13 mothers, and 10 fathers in the non-attempter group. Independent samples *t*-tests showed no significant differences in BSDQ scores based on whether or not the adolescent made a banking attempt (see Table 2). However, examination of Cohen’s *d* effect sizes revealed moderate effect sizes when comparing attempter and non-attempter mothers (*M*_attempter-mothers_ = 6.13 vs. *M*_non-attempter-mothers_ = 5.68; *d* = 0.45), as well as fathers (*M*_attempter-fathers_ = 6.44 vs. *M*_non-attempter-fathers_ = 6.17; *d* = 0.44). There was a negligible effect for adolescents across attempter and non-attempter groups (*M*_attempter-adolescents_ = 5.76 vs. *M*_non-attempter-adolescents_ = 5.68; *d* = 0.08).

### 3.3. Qualitative Results

Thematic analysis of mother, father, and adolescent transcripts from the non-attempter group (24 total participants representing 15 families) revealed three themes: (1) potential for future decisional regret, (2) acceptance of the decision and any future implications, and (3) satisfaction with the decision. Thematic analysis of mother, father, and adolescent transcripts from the attempter group (58 total participants representing 24 families) revealed only one theme: (1) satisfaction with the decision.

#### 3.3.1. Non-Attempter Group Themes

##### Potential for Future Regret

When asked about how they feel about their banking decision, several participants from non-attempter families described the potential for future regret. This was often expressed as recognizing the infertility risk and the potential disappointment that could result if biological parenthood was unattainable. A few participants noted that they actively regretted their decision, stating that they wish they had made a different decision. An additional few noted that they had serious doubts about their decision given the impact that such a decision has on their future.

“Iffy on it... If I can’t, then I’ll be probably be a little upset.” (17-year-old, osteosarcoma)

Most participants, however, noted regret only in the context of the future. In these cases, regret was acknowledged in a more prospective way, noting that there could be a change in perspective through time. This was often seen as something that could occur later despite current attitudes about the decision:

“There’s always a chance (of him) saying, ‘Oh I wish back when I was 12 I had done that’ or ‘Boy I wish you guys would have talked me into doing that back when I was 12 because I feel differently now of course.” (Father of a 12-year-old, acute myeloid leukemia)

Others discussed regret in terms of a future partner’s perspectives, noting that although the son could feel satisfied with the decision in the present, that perspective could change if a future partner desires a biological child:

“The only thing I’ve worried a little bit about is how a future partner might look at his decision he made when he was 12.” (Father of a 12-year-old, Ewing’s sarcoma)

##### Acceptance of the Decision

Many participants described a sense of acceptance with the decision not to bank. Regardless of the future implications of the decision, many participants noted that the decision had been made and that there was little they could do about it:

“In the future if he regrets that choice, there’s not a whole lot we can do about that…this decision was right for the time, and you can’t take it back.” (Mother of a 17-year-old, osteosarcoma)

Some participants discussed additional considerations that contributed to their sense of acceptance. Some participants discussed prioritizing health over FP at diagnosis, stating that they were okay with the decision because health is paramount.

“I don’t care…as long as he’s healthy, I could care less. His health is the priority.” (Mother of a 16-year-old, medulloblastoma)

Other participants discussed their decision in terms of religion. These families accepted their decision and related future implications because they viewed the outcome as being part of their destiny/God’s will.

“He’s a pretty faithful guy, so I think in the end if it does turn out that he cannot have biological children, I think he would probably be like, “Well, that was God’s decision,” and he would just rest with that...We’re Christian, and he prays, and he does hold faith in a higher being. So, I know that he’s put a lot of these issues into that faith.” (Mother of a 16-year-old, acute lymphoblastic leukemia)

Participants also accepted their decision by noting the possible use of alternative routes to parenthood in the case of infertility (adoption/fostering).

“I’m hoping that it ultimately doesn’t matter…that I’ll still be able to have my own kids, but if it doesn’t happen, I’m always open for adoption.” (19-year-old, Burkitt’s lymphoma)

##### Satisfaction with the Decision

Several participants felt good about their decision and described a sense of satisfaction and confidence with the choice not to bank. They noted that they were comfortable and did not have any regrets, nor did they acknowledge a possibility for future regrets:

“I feel really good about it…I think overall we’re very confident in where we’re at.” (Father of a 13-year-old, acute myeloid leukemia)

Some participants described feeling satisfied because they were interested in adoption and fostering and found these family building options to be meaningful in the case of infertility or even in general:

“Seeing him interact with his adopted friend they talk very openly about adoption all the time...he wants everyone to adopt so again…that’s something that he’s passionate about. So, I think it makes sense, and I think he’ll feel good about it.” (Mother of 12-year-old, acute myeloid leukemia)

Other participants discussed feeling satisfied with the decision not to bank because it was the son’s decision to make. Therefore, regardless of the outcome, they would be happy with the choice. Some participants also mentioned a sense of pride with their son’s autonomous role in decision making:

“I feel that I made the right decision, and I’m satisfied with my decision. I don’t regret anything…I think (my parents) probably feel satisfied knowing that I’m satisfied with my decision.” (12-year-old, Ewing’s sarcoma)

#### 3.3.2. Attempter Group Themes

##### Satisfaction with the Decision

Almost all participants from the attempter group reported feeling satisfied with their decision to bank. Despite occasionally noting the financial burden of storing a sample and the potential emotional toll of utilizing the sample in the future, participants overwhelmingly discussed their satisfaction with the choice, both currently and in the future.

“I feel like it was a good decision…I don’t think I will regret it at all. I think it was a really good idea.” (18-year-old, lymphoma)

“I feel good about that…I’m just happy that he could see and understand…what we were saying…I think that he will feel really good about it.” (Mother of a 19-year-old, Burkitt’s lymphoma)

Many participants attributed satisfaction with this decision to a personal preference for biological (grand) parenthood, emphasizing this option for family building over other alternatives. To these participants, sperm banking was the best option because it allowed for the opportunity to pursue biological parenthood, which was highly valued.

“I’ll still feel good because I know I’m getting me a grandbaby…I feel good knowing that I will have something that is made by him.” (Mother of a 13-year-old, Hodgkin’s lymphoma)

Additionally, participants discussed satisfaction with the decision because it not only provided their son with the opportunity for biological parenthood but it also provided a potential future partner with that opportunity. Participants noted that a future partner may want a biological child, which could complicate relationships or cause their son to feel distressed if facing infertility.

“If I’m older and I’m married…and I have a wife that wants to have kids, obviously I’m going to use the sperm thing.” (19-yeard-old, Burkitt’s lymphoma)

Similarly, participants noted satisfaction with banking because it generally gave them an alternative and acted as a safeguard in case infertility ensues.

“It’s just something we’re doing, kind of like insurance…you hope you never have to use it, but at the same time, it’s there in case you do.” (Father of a 16-year-old, rhabdomyosarcoma)

Others noted a sense of satisfaction due to adolescent’s autonomous role in making the decision and described feeling proud/happy of the adolescent’s maturity to make such a difficult decision given the overwhelming nature of the situation.

“I feel good,‘cause I know that makes my son happy, ‘cause he knows he has these chances of having biological children.” (Mother of a 15-year-old, rhabdomyosarcoma).

### 3.4. Mixed Methods Results

Integrating quantitative findings with qualitative findings, similarities emerged in the reported satisfaction. High quantitative satisfaction scores on the BSDQ were reflected in participant interview transcripts, as both groups reported feeling satisfied with the decision and discussed factors that contributed to this satisfaction. Despite this convergence, qualitative reports in non-attempter families of the potential for future regret and acceptance with the decision were not captured in the quantitative data (see Table 4).

## 4. Discussion

Overall, we found that adolescents, mothers, and fathers from both attempter and non-attempter groups had relatively high decisional satisfaction scores approximately one month after diagnosis and their FP decision. However, examination of qualitative responses revealed that individuals from non-attempter families also expressed the potential for future regret or the acceptance of future risk for infertility. Taken together, the findings underscore the importance of a mixed methods approach in capturing the holistic experiences of families facing FP decisions. In the short term, it may be important to assess both current decisional satisfaction and the potential for future decisional dissatisfaction, in order to optimize quality of life, elucidate risk for later distress, and target psychosocial support accordingly.

High decisional satisfaction was observed across our whole sample, in both attempter and non-attempter groups. Quantitatively, decisional quality scores were high and statistically similar between attempt groups, but effect sizes suggest that mothers and fathers in the attempter group were more satisfied than those in the non-attempter group, with no differences in effect sizes for adolescents’ quantitative decisional satisfaction. As a participant type, fathers reported higher scores on the BSDQ compared to adolescents. Additionally, both attempter families and non-attempter families reported satisfaction qualitatively; however, satisfaction was overwhelmingly reported among attempter families, whereas it was reported at lower rates by non-attempter families. These differences parallel the literature on long-term decisional satisfaction, suggesting that decisional satisfaction may be lower among those who decide to forgo FP [4,6,19].

Research has shown that standardizing approaches to fertility counseling and integration of decision tools improves decisional satisfaction and quality of life [30,32]. In a study in adult cancer populations, short-term, low decisional regret was reported among those who received a decisional aid in addition to fertility counseling [39,40]. Long-term decisional satisfaction was also high following the use of both fertility counseling and a decision aid [32]. Adolescents have been known to prefer family support over physician support when making decisions about sperm banking [41]. Family-centered decision tools and counseling approaches could be especially important considering that family functioning and support can act as a buffer against the impact of stress on decisional conflict [42]. Additionally, this could be helpful for younger adolescents who may have a limited capacity to engage in future-oriented thinking [17,18].

Our findings and those of previous studies suggest that both fertility counseling and use of a decision tool (i.e., the FAST) [31] may have contributed to the high decisional satisfaction reported in both groups. Participants in this study noted benefits of this tool with regard to prompting deeper thinking and influencing conversations about FP [43]. It is plausible this facilitated their FP decision making and reduced decisional conflict. The link between FP counseling, decision tools, and both satisfaction and quality of life highlights the importance of dedicated FP programs [10], reproductive health communication training for providers [43], and tailored family-centered decision tools geared for different ages and developmental stages in adolescent cancer populations.

Consistent with research on the emergence of regret in survivorship [4,6,19], participants in the non-attempter group noted a potential for regret in the future. The potential for future regret was often noted in addition to current satisfaction or acceptance, suggesting that current regret may not be actively experienced but could appear later in treatment or in survivorship and could impact future quality of life [21,22]. Considering that the previous literature reported regret among families who decided not to bank [4,6,19], it is possible that a short-term examination of decisional satisfaction may not reflect long-term outcomes, as it may be too soon for families to reflect on their decision or for it to be immediately relevant. A new cancer diagnosis can be emotionally and physically straining [44]; therefore, this decision may not be on the forefront of their minds. Research has shown that reproductive health and family building is often a topic that adolescents have not thought about prior to receiving an FP consult [43]. Given that these topics become more salient in adulthood, coupled with developmental limitations in future-oriented thinking [18], it is unsurprising that adolescents may continue to feel satisfied with their decision one month following a diagnosis. However, in the long term, perspectives could change, especially among those who decided not to bank, as this group has been observed to cite poor decision quality in the previous literature [23]. Taken together, our findings suggest the importance of continued care and follow-up across ages and the stages of development, especially among those adolescents who did not bank, to identify any potential signs of regret and need for support.

The non-attempter group also discussed a sense of acceptance with the decision and future implications. Many participants felt that there was nothing they could do to change a decision that they made in the past; consequently, participants eased their worries by thinking about options/factors that make the decision and its implications more acceptable. For some, this took the form of wanting to prioritize health over treatment delays. Concerns about delaying treatment are a common barrier to FP, highlighting the importance of early discussion and close collaboration with providers to reassure families that pursuing FP will not impact their health outcome [41]. Additionally, participants considered their decision based on their religious views and the availability of alternative parenthood options (adoption/fostering), which is consistent with other decisional satisfaction studies [45,46,47]. Thus, acceptance may be a positive strategy for families to utilize following a decision, as positive affect has been connected to lower decisional regret in other medical contexts [48]. If regret should arise, clinicians could foster acceptance among adolescents or young adults to help them cope with their decision and encourage consideration of these alternatives.

### Limitations

There were several limitations to this study. First, our sample was primarily white, and data were collected from a small sample at a single pediatric institution, with an established fertility program, and following the use of an FP values clarification tool. Differences in decisional satisfaction may be noted in populations that did not receive pre-treatment fertility counseling/FP decision tools. Collecting data from multiple institutions may highlight a more diverse cohort in terms of demographic characteristics, as well as factors relating to decisional satisfaction. Our small sample had limited statistical power, and a larger sample might show significant differences in decision quality scores between those who did and did not attempt FP, as well as allowing for in-depth examination of concordance within families as well as the moderating effect of the infertility risk level on associations between banking attempt and decisional satisfaction. Secondly, both study visits were conducted within a short time span after a cancer diagnosis, which is a sensitive and overwhelming time for these families. Further, quantitative and qualitative questions were thematically similar but not identical, meaning it is possible that the findings may have been different had measures been more consistent. Thirdly, although decisional satisfaction included multiple domains (regret, satisfaction, involvement, “right for you”, information, and time), each domain was a single item, meaning we were inadequately powered to assess which aspect of decisional satisfaction could have driven differences between attempter groups. Finally, this study only collected data on short-term FP decisional satisfaction. Longitudinal research is needed to explore FP-related decisional satisfaction in adolescents as they approach young adulthood.

## 5. Conclusion

Quantitively, short-term decisional satisfaction was high regardless of the banking decision among families of adolescent males newly diagnosed with cancer. However, the qualitative findings suggest that the experiences of families who did not bank may be more nuanced, as some participants discussed a potential for future regret. Standardized tools are needed to better capture these aspects of decisional satisfaction and examine associations with later outcomes, such as quality of life. Further, longitudinal research and clinical assessments at different ages and developmental stages are important to identify changes in satisfaction, acceptance, and regret and manage any fertility-related distress that may occur across the treatment continuum.

## Figures and Tables

**Table 1:** Sample characteristics.

Sample Characteristics	Adolescents		Mothers		Fathers	
	*n*	%	*n*	%	*n*	%
Race						
White	27	77%	30	88%	19	86%
Black	2	5%	3	9%	-	--
Native Hawaiian/Pacific Islander	4	11%	1	3%	1	5%
Other	2	6%	-	--	2	9%
Ethnicity						
Non-Hispanic/Latino	30	86%	34	100%	20	91%
Hispanic/Latino	1	3%	-	--	1	5%
Relationship Status						
Single/Separated/Divorced	23	66%	7	21%	5	23%
Married/Engaged/In a Serious Relationship	8	23%	27	79%	17	77%
Religion						
Christian	19	54%	28	82%	13	59%
Catholic	3	9%	-	--	1	5%
Jewish	1	3%	1	3%	1	5%
Muslim	-	--	1	3%	-	--
Agnostic	1	3%	1	3%	3	14%
None	5	14%	2	6%	3	14%
Other	2	6%	1	3%	1	5%
Income						
Less than USD 25,000			3	9%	1	5%
USD 25,000–USD 49,999			7	21%	2	9%
USD 50,000–USD 74,999			6	18%	4	18%
USD 75,000–USD 99,999			8	24%	5	23%
USD 100,000–USD 149,999			4	12%	4	18%
USD 150,000 or more			4	12%	6	27%
Unsure			2	6%		
Level of Education						
Some High School			2	6%		
High School Diploma or GED			6	18%	2	9%
Some College, No Degree			6	18%	9	41%
Associate Degree			5	15%	2	9%
Bachelor’s Degree			11	32%	4	18%
Graduate or Professional Degree			3	9%	4	18%
Other			1	3%	1	5%
Diagnosis Type						
Brain or Spinal Cord Tumor	4	11%				
Leukemia	8	23%				
Lymphoma	12	34%				
Solid Tumors	10	29%				
Other	1	3%				
Risk for Infertility						
Minimally Increased Risk	13	37%				
High Level of Increased Risk	22	63%				

**Table 2:** Comparison of decision quality scores between attempter and non-attempter families.

Comparison of Decision Quality Scores between Attempter and Non-Attempter Families ^a^	Attempters		Non-Attempters		Composite Score				
	M	SD	M	SD	M	SD	*t*-Test	*p*	*d*
Patient BSDQ Score	5.76	0.97	5.68	1.03	5.79	0.98	−0.23	0.821	0.08
Mother BSDQ Score	6.13	0.72	5.68	1.02	5.98	0.94	−1.18	0.255	0.45
Father BSDQ Score	6.44	0.61	6.17	0.61	6.37	0.61	−1.02	0.322	0.44

^a^ Additional independent samples *t*-tests were examined to compare single items on the BSDQ (i.e., regret, satisfaction, involvement, “right for you”, information, and time) by banking attempt. Significant differences were found for mothers’ regret (*M*_attempter-mothers_ = 7.00 vs. *M*_non-attempter-mothers_ = 6.10; *t*(11) = −2.42, *p* = 0.034), mothers’ satisfaction (*M*_attempter-mothers_ = 6.59 vs. *M*_non-attempter-mothers_ = 5.25; *t*(13.57) = −2.36, *p* = 0.034), and adolescents’ information (*M*_attempter-adolescents_ 5.08 vs. *M*_non-attempter- adolescents_ = 6.82; *t*(28.84) = 3.42, *p* = 0.002). No other significant differences were found by banking attempt.

**Table 3:** Comparison of decision quality scores between adolescents, mothers, and fathers.

Variable	n	M	SD	*t*-Test	*df*	*p*
Adolescents vs. Mothers				−1.61	31	0.119
Adolescents	32	5.79	0.98			
Mothers	32	5.97	0.94			
Adolescents vs. Fathers				−2.26	19	0.036 *
Adolescents	20	5.93	0.90			
Fathers	20	6.37	0.61			
Mothers vs. Fathers				−1.31	17	0.209
Mothers	18	6.18	0.95			
Fathers	18	6.44	0.52			

* *p* < 0.05.

**Table 4:** Joint display of decision quality among non-attempters and attempters.

Domains	Qualitative Investigation	Frequency (%)	Quantitative Investigation *	Mixed Methods Interpretation
**Non-attempter group**				Quantitively, short-term decisional satisfaction was high regardless of banking decision. However, qualitative findings suggest experiences of families who did not bank may be more nuanced, as participants in this group reported a potential for future regret as well as a sense of acceptance with their decision.
**Potential for** **regret**	*“My biggest fear is that he would regret it later.”* (Mother of a 12-year-old, acute myeloid leukemia) “I mean obviously if we get 15 years down the road or whatever and he and his future wife are having trouble conceiving a child, there probably is gonna be a time where we’re gonna look at each other and go, did we do the right thing?” (Father of a 13-year-old, acute myeloid leukemia) “I feel like it could have been a good thing but just at that time it wasn’t…something we really could have done…(how I feel in the future) dependss if I’m fertile or not...” (14-year-old, osteosarcoma)	**Overall: 50%** Adolescents: 36% Mothers: 45%, Fathers: 44%	BSDQ Means: Adolescents: *M* = 5.68 Mothers: *M* = 5.68 Fathers: *M* = 6.17	
**Acceptance**	“I’m sure when I see him active and healthy and himself you know that would help me a lot that I didn’t delay (treatment)…if we delayed it, he would probably not be here.” (Mother of a 13-year-old, germ cell carcinoma) “I agree with him…if it’s God’s will, I guess…He can always, I guess adopt.” (Father of a 20-year-old, acute lymphoid leukemia) “Probably wish I had done it if I can’t have kids but what are you gonna do? It’s over with.” (16-year-old, acute lymphoblastic leukemia)	**Overall: 60%** Adolescents: 50% Mothers: 64%, Fathers: 67%		
**Satisfaction**	“Completely comfortable with the decision. Completely good with it…whatever his decision was, that’s his choice.” (Mother of a 12-year-old, Ewing’s sarcoma) “He would kind of like to help somebody who needs help, somebody who might need adopted, like his friend, and some other people he’s known.” (Father of a 12-year-old, acute myeloid leukemia) “I think I’m very satisfied with it. I think it was the right decision for me. Maybe others would choose something else but for me it was the best decision for me.” (17-year-old, chronic myelocytic leukemia)	**Overall: 40%** Adolescents: 40% Mothers: 36%, Fathers: 44%		
**Attempter group**				
**Satisfaction**	“It was 100% good…I was happy that he (banked) and I am very happy the topic came up.” (Mother of an 18-year-old, CNS tumor) “If he’s like alright and he finds the girl…and they start their life together then…it all works out and it would be like again no brainer good” (Father of a 16-year-old, rhabdomyosarcoma) “I think my family might appreciate it when I’m older if I actually am not able to have kids and I think that they might be thankful that I did it because then I’ll actually be able to have my own biological child.” (17-year-old, nodular sclerosing Hodgkin’s disease)	**Overall: 93%** Adolescents: 96% Mothers: 91%, Fathers: 92%	BSDQ Means: Adolescent: *M* = 5.76 Mothers: *M* = 6.13 Fathers: *M* = 6.44	

* *t*-tests comparing participants in non-attempter vs. attempter groups were non-significant (*p* < 0.05).

## Data Availability

The data presented in this study are available upon request from the corresponding author.
